# Clinical Relevance of Total HIV DNA in Peripheral Blood Mononuclear Cell Compartments as a Biomarker of HIV-Associated Neurocognitive Disorders (HAND)

**DOI:** 10.3390/v9110324

**Published:** 2017-10-31

**Authors:** Vurayai Ruhanya, Graeme B. Jacobs, Richard H. Glashoff, Susan Engelbrecht

**Affiliations:** 1 Division of Medical Virology, Department of Pathology, Faculty of Medicine and Health Sciences, Stellenbosch University, Francie van Zijl Avenue, P.O. Box 241, Cape Town 8000, South Africa; graeme@sun.ac.za; 2 Department of Medical Microbiology, College of Health Sciences, University of Zimbabwe, P.O. Box A178, Avondale Harare 00263, Zimbabwe; 3 Division of Medical Microbiology and Immunology, Department of Pathology, Faculty of Medicine and Health Sciences, Stellenbosch University, Francie van Zijl Avenue, P.O. Box 241, Cape Town 8000, South Africa; rglas@sun.ac.za; 4 Division of Medical Microbiology and Immunology, National Health Laboratory Service (NHLS), Tygerberg Business Unit, P.O. Box 241, Cape Town 8000, South Africa; 5 Division of Medical Virology, National Health Laboratory Service (NHLS), Tygerberg Business Unit, P.O. Box 241, Cape Town 8000, South Africa

**Keywords:** HAND, HIV DNA, qPCR, biomarker

## Abstract

The pathogenesis of HIV-associated neurocognitive disorders is complex and multifactorial. It is hypothesized that the critical events initiating this condition occur outside the brain, particularly in the peripheral blood. Diagnoses of HIV-induced neurocognitive disorders largely rely on neuropsychometric assessments, which are not precise. Total HIV DNA in the peripheral blood mononuclear cells (PBMCs), quantified by PCR, correlate with disease progression, which is a promising biomarker to predict HAND. Numerous PCR assays for HIV DNA in cell compartments are prone to variation due to the lack of standardization and, therefore, their utility in predicting HAND produced different outcomes. This review evaluates the clinical relevance of total HIV DNA in circulating mononuclear cells using different published quantitative PCR (qPCR) protocols. The rationale is to shed light on the most appropriate assays and sample types used to accurately quantify HIV DNA load, which predicts severity of neurocognitive impairment. The role of monocytes as a vehicle for trafficking HIV into the CNS makes it the most suitable sample for determining a HAND associated reservoir. Studies have also shown significant associations between monocyte HIV DNA levels with markers of neurodamage. However, qPCR assays using PBMCs are cheaper and available commercially, thus could be beneficial in clinical settings. There is need, however, to standardise DNA extraction, normalisation and limit of detection.

## 1. Introduction and Brief Pathology of HAND

Highly active antiretroviral therapy (HAART) has reduced the prevalence of AIDS related opportunistic infections. Consequently, efforts are being directed at managing long-term HIV complications such as central nervous system (CNS) diseases, which have continued to affect patients, despite adequate virological control [1]. Some of the most frequent complications in patients with HIV-1 are HIV-associated neurocognitive disorders (HAND), which involve impairment or disruption of neurocognitive functioning [2]. It is estimated that 20–69% of all HIV-1 cases have neurocognitive disorders [3,4]. There is a wide range of disorders covered in the description of HAND according to the 2007 Frascati criteria of categorising severity of neurocognitive impairment [5]. Dementia associated with HIV, HAD, is the most severe form of HAND. Mild neurocognitive disorder (MND) is characterised by minor neurocognitive impairment and asymptomatic neurocognitive impairment (ANI) is characterised by two or more cognitive abilities with no functional impairment.

**Figure 1:**
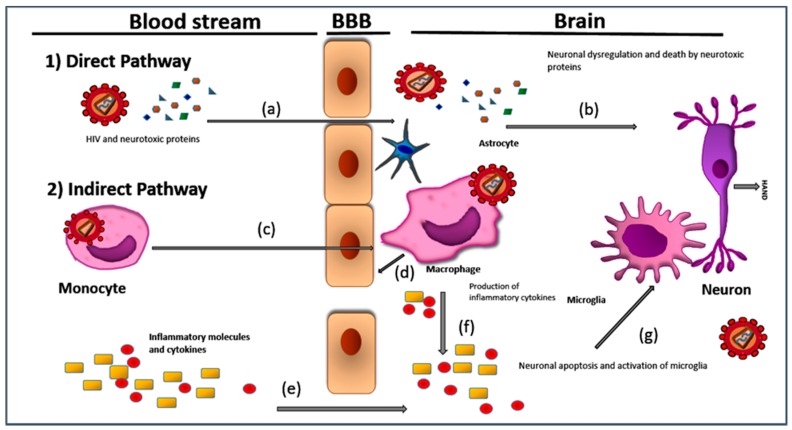
Mechanism of neuropathogenesis. Two pathways involved shown by arrows: (1) Direct pathway caused by HIV and released HIV proteins. (2) Indirect pathway involving secretion cytokines. (**a**) Virus particles and viral proteins shed and cross Blood brain barrier (BBB). (**b**) Neural injury caused by direct viral infection and dysregulation by viral proteins. (**c**) Infected monocyte infiltrating BBB. (**d**) Release of cytokines from infected monocytes contributing to disruption of BBB. (**e**) BBB become more permeable to cytokines present in the periphery. (**f**) More cytokines released into the brain and (**g**) cytokines disrupt normal functioning ultimately leading to neuronal apoptosis resulting in different forms of HIV associated neurocognitive disorders (HAND).

The precise mechanism of the neuropathogenesis of HAND is unknown, but studies have shown that HAND is likely determined by a complex interaction between viral factors, cellular targets and the immune response. Two models have been proposed to explain neurodegradation and the development of symptomatic HAND; namely, the direct and indirect pathways (Figure 1). Both initially require HIV-1 infection of the perivascular macrophages and microglia in the brain. According to the “Trojan horse” hypothesis, HIV-infected perivascular monocytes/macrophages traffic HIV from the peripheral blood to the CNS, leading to the infection of the resident microglial cells [6].

The direct pathway proposes that the viral proteins (Env gp120, Tat and Vpr), released from HIV-infected monocyte-derived cells, can cause neuronal death by direct interaction with neurons. Among the direct effects of viral proteins on neurons is neuronal depolarization by direct excitation independent of synaptic interactions, N-methyl-D-aspartate (NMDA) receptor dysregulation and disruption of calcium homeostasis in the case of Tat [7,8]. The indirect pathway suggests that neuronal death is mediated by inflammatory cells targeting HIV proteins released by infected cells. Infected or activated macrophages release a variety of soluble cytokines and chemokines, which are pro-inflammatory, including IL-6, GM TNF-α-CSF and IL-1β, which eventually leads to neuronal apoptosis [9,10].

Since there is no standard protocol to assess the condition optimally, HAND is difficult to diagnose. The screening and diagnosis for HAND relies on multiple clinical and neuro-psychometric methods, such as the World Health Organisation (WHO) Auditory Verbal Learning Task (WHO-AVLT) and the Revised Brief Visual Memory Task (BVMT-R) for learning efficiency [11,12]. These methods have been used to define the scope and severity of HAND. However, there is need to compliment clinical and neuropsychometric assessments with HIV-specific biological markers underlying symptomatic HAND. The availability of reliable biomarkers would help to diagnose patients and bring a better understanding of neuropathogenesis and therapeutic strategies to HAND [13]. Although many proposed potential biomarkers for HAND have been described, there is a lack of sensitivity and some of them are not HIV specific [14]. They include markers of monocyte activation such as soluble CD163 and CD14 (sCD163 and sCD14) [15], cytokine and chemokine receptors as well as markers of neural injury and glial dysfunction [1,16,17,18]. They do not distinguish between the severity and HAND subtype. No longer closely associated with neuropsychometric performance are HIV specific markers such as plasma CD4+ T-cell count and viral load [19] in this era of HAART, where prevalence of HAND in aviremic patients is common. Peripheral blood mononuclear cells (PBMCs) containing HIV DNA has been linked to HAND progression in virally suppressed patients and therapy naïve patients [11,20]. This review summarises recent developments on the potential use of total peripheral blood mononuclear cell-associated HIV DNA as a marker of HAND, focusing on PCR quantification assays. The rationale is to highlight the potential clinical utility of this biomarker for HAND, considering the roles played by different mononuclear cell compartments (lymphocytes compared to monocytes) or unfractionated PBMCs in the proposed HAND pathogenesis models, bringing into context the suitability of different samples used in different PCR assays. A careful analysis of published PCR methods regarding sample type, sample loading quantity and quality, normalisation, robustness, accuracy, sensitivity and specificity is required before total peripheral HIV DNA is used as a biomarker for HAND in clinical settings.

## 2. Latency and Different Forms of HIV DNA in the Periphery

Immune cells are infected by HIV through CD4 receptors, and the co-receptors CCR5 and/or CXCR4. The primary target cell is the CD4+ T lymphocyte. Following cellular infection, viral genetic material (RNA) is then reverse transcribed into HIV DNA that becomes integrated into host DNA or might exist as non-integrated linear and circular forms (1-LTR and 2-LTR circles) [21]. A subset of these infected immune cells may become memory cells that harbour HIV DNA without the production of virus (i.e., latently infected) [22,23]. This latency state may be disrupted if the cells are re-stimulated/activated or when treatment is interrupted [21,23]. The HIV DNA is persistently stored in a stable form in lineages of memory T lymphocytes and is protected from biochemical decay [23]. Monocytes may be directly infected or may be infected following phagocytic uptake of infected lymphocytes and/or free virions. Infection may occur in the blood or at the bone marrow level [24]. Antiretroviral therapy (ART) reduces HIV RNA particles in circulating blood to undetectable levels, but it cannot eliminate HIV DNA infecting white blood cells [22]. Latently infected cells are disseminated throughout the body, but are differentially concentrated in the lymphoid tissues [25]. Blood is the most accessible tissue, but not the true representative medium for measuring the latent reservoir [26]. Furthermore, the frequency of HIV infected cells is higher in the gut associated lymphoid tissue than in the blood [27,28,29]. Outlined earlier, the systemic HIV DNA reservoir trafficking to the CNS with activated cells (monocyte/macrophage lineages) causes activation-induced neurodamage [30,31].

The easy accessibility of peripheral blood as a clinical specimen makes it more feasible to investigate the contribution of peripheral HIV DNA reservoirs to pathogenesis of neurocognitive impairment [24,28]. Peripheral HIV DNA is an independent predictor of HIV disease irrespective of combined antiretroviral therapy and was widely used in both adult and paediatric cohorts [32,33,34,35]. The HIV DNA in peripheral blood has also been used as a prognostic marker of disease progression, especially in suppressed patients where it is the only biomarker of viral activity [35]. Therefore, it is possible to investigate the role of peripheral HIV DNA in neuropathogenesis by evaluating the association between the clinical categories of HAND (ANI, MND and HAD) and the quantities of HIV DNA. Available assays can quantify both total and unintegrated HIV DNA forms (linear, 1-LTR and 2-LTR) [36,37].

## 3. What Samples Can Be Used to Measure HIV DNA as a Biomarker for HAND?

Assays to quantify HIV DNA by qPCR have utilised both PBMCs and sorted cell subsets as clinical samples, but choices must be made on the most appropriate samples to collect and process [38]. The appropriate clinical specimen in HIV DNA qPCR should give a true size on the peripheral HIV-1 DNA pool and its composition [36], which enables clinical diagnosis and monitoring disease progression [39,40]. It is important to decide on the type of sample and form of DNA to use as a marker to define and monitor the progression of HAND, considering the size of the HIV reservoir in the sample and its role in the pathology of the disease. An appropriate clinical specimen gives information which reflects on the condition of the disease by correlating with findings from clinical assessments [41]. The choice of the sample should consider the stage of HIV disease as it is known that there are differences between early HIV disease and advanced stages of infection with regard to shift in tropism [42]. Advanced stages of HIV diseases shows depletion of CD4+ T lymphocytes, while there is both expansion and increased turnover of peripheral monocytes, including CD14+/CD16+ subsets known to be associated with increased susceptibility to HIV infection [43]. Therefore, investigations should consider the clinical stage of neurocognitive impairment/severity of HAND since there is likely to be differences in quantities of PBMC subsets and the composition of different forms of HIV DNA in the cells. Sample type is very critical because it has implications, first on the quantity and forms of DNA in the cell type and, second, the role played by the cell type in HAND pathogenesis. All three forms of HIV DNA have been detected in brain tissues of patients having AIDS dementia [44]. Some studies have measured HIV DNA quantities using the whole blood, while others have used PBMC and found good correlation between the two samples [34,36]. To consider peripheral HIV DNA load as a reliable virological marker of HAND in clinical settings, a careful assessment of PBMC and its cell subsets as appropriate samples is required.

### 3.1. Unfractionated PBMC

Peripheral blood mononuclear cells constitute the cellular part of the blood containing cells with a round nucleus, namely monocytes, T cells, B cells, Natural killer cells (NK) and dendritic cells [45]. Peripheral blood mononuclear cells (PBMCs) are involved in many immune-related diseases and there is growing interest to use them as surrogate markers of several diseases [46,47]. These PBMCs can be obtained relatively easily from routinely collected blood samples without analytical difficulties [48]. Thus, there is a growing interest to use PBMCs diagnostically as surrogates for direct sampling of sites of infection and to investigate other disease processes such as HIV-associated neurocognitive impairment [32,49]. It has been demonstrated that HIV DNA levels in PBMCs correlate with antiretroviral therapy efficacy, suggesting that DNA quantitation is a useful tool to monitor the decay of the HIV reservoir, especially when plasma viremia is undetectable [50].

Significant positive correlations between levels of HIV DNA measured in PBMC and CSF cell pellets has been reported in HAND patients, showing that there was a link between peripheral blood HIV reservoirs which influence CNS HIV DNA quantities [51]. Measurement of HIV DNA in unfractionated PBMCs using PCR is the simplest way of quantifying the reservoir in the peripheral blood [52]. The PBMC HIV DNA levels have been linked to cortical and subcortical grey matter atrophy in patients on HAART with undetectable viral load, and it was also observed that HIV DNA in PBMCs in HAD was 20 times higher than in uninfected individuals [53,54]. Since brain biopsy and CSF are not a clinically feasible option in HAND studies, utility of PBMCs is imperative. The quantity of HIV DNA in unfractionated PBMCs represents the true size of the peripheral HIV reservoir, in fact, because HIV DNA differentially distributed in all cell subsets are considered in PCR assays [31]. However, some studies on the association between HIV DNA in unfractionated PBMC and HAND did not show significant correlation [53]. Therefore, there is need to analyse the role of separate peripheral mononuclear cell subsets (Lymphocytes/monocytes) in neuropathogenesis.

### 3.2. Lymphocytes

The primary target for HIV infection in the peripheral blood are CD4 T lymphocytes, which makes them the predominant cell type harbouring HIV-1 in the peripheral blood of infected individuals [55,56,57]. The majority of HIV DNA is contained within memory (CD57−CD4+) T cells [57]. Research has also found that all forms of HIV DNA persist in resting CD4 cells, suggesting that there is replenishment of the reservoir, either by reactivation or by infection [21,57]. It has also been demonstrated, both in vitro and in vivo using animal models, that cytotoxic T lymphocytes can be infected by HIV-1 (Stanley et al., 1993). It is thought that the infection takes place during the peripheral interaction between CD4 and CD8 as part of an immune response, thereby allowing direct transmission of HIV-1 to the CD8 lymphocytes [27,56]. Using CD4 and CD8 naive and memory cell populations separated from PBMCs of HIV-seropositive individuals, frequencies of infected cells ranged from 30 to 670 proviral copies/10^6^ CD4+ lymphocytes and from 8 to 500/10^6^ CD8 lymphocytes [56]. The reservoir of HIV in CD4+ and CD8 lymphocytes is of clinical significance due to their important role in HIV pathogenesis, as well as their routine application in diagnostic settings as markers of HIV infection. Using qPCR targeting the HIV *gag* region, it was shown that memory CD57−CD4+ T cells were always more frequently infected (10–10,000 copies of *gag* DNA/10^5^ cells) than effector CD57+CD4+ T cells [58]. The quantity of infected memory cells in this study also correlated with plasma viral load, which is a virological marker for monitoring and predicting HIV infection. However, with regard to HAND, there are limited data on correlation studies linking peripheral lymphocyte HIV DNA levels and severity of neurocognitive impairment [42,51]. Investigations using ART naïve participants with mild to moderate neurocognitive impairment, lymphocyte HIV DNA levels were shown to be significantly associated with HAND independent of plasma viral load and CD4+ count [42,54]. There is need for more data on association studies between peripheral lymphocyte HIV DNA reservoirs and HAND, since this is the preferential target of HIV infection [57]. Activated CD4+ T lymphocytes support viral replication and resting cells support establishment of a latent reservoir [58,59]. The result of HIV replication in activated lymphocytes is the production of different forms of HIV DNA (linear, 1-LTR and 2-LTR) and resting cells predominantly constitute integrated HIV DNA, both of which have shown to have clinical consequences in the brain HAND patients [60,61]. Therefore, this cell subset can represent the true size of the reservoir with different forms of HIV DNA.

### 3.3. Monocytes

There are two major types of monocytes in the blood, classical monocytes that have a high expression of CD14+ cell surface receptor (CD14++ monocytes) and non-classical, pro-inflammatory monocytes with a low CD14+ marker, but with an additional CD16+ marker (CD14+CD16+ monocytes) [62]. However, it has been reported that there is heterogeneity in CD14+CD16+ monocytes which resulted in subdivision of the pro-inflammatory monocytes into intermediate (CD14++CD16+) and non-classical (CD14+CD16++) monocytes [63]. Analysis by cellular subsets revealed that differences in cognitive categories are related to the amount of HIV DNA within inflammatory monocytes (CD14+CD16+) as compared to non-inflammatory monocytes (CD14+CD16−) or the CD14− fraction, which includes the lymphocytes [53,64]. Using a series of immunohistochemical markers, studies have demonstrated that CD14+CD16+ monocytes derived from the periphery accumulated in the perivascular region of the brain in patients with dementia [65]. The infection of monocytes by HIV is thought to arise from virus released from re-activated infected CD4+ T cells in the blood or infection at the bone marrow level [27,62,66].

The CD14+CD16+ monocyte subset in the peripheral blood constitutes only 5–10% of peripheral blood monocytes in healthy individuals, but the percentage increases to approximately 40% in HIV infected people [67]. Both in vitro and in vivo studies demonstrated that CD14+CD16+ monocytes express high amounts of the co-receptor CCR5, which facilitates their infection. It has also been observed that cells of the monocyte lineage are not susceptible to the cytopathic effects of HIV and, once they are infected in the periphery, they produce infectious virions as they enter the CNS and when they differentiate into macrophages [27]. Monocytes circulate in the blood for up to three days before they enter tissues [27,62,66,67]. Since monocytes are only present for 1–3 days in the blood, the presence of viral DNA within these cells indicate recent infection [68]. Monocytes isolated from blood of HIV patients were shown to contain unintegrated circular viral DNA, suggesting transcriptionally active rather than latent infection [69]. However, it has been shown that blood purified monocytes contain HIV DNA over time in both treated and untreated patients [7,27,70,71].

Monocytes circulate freely and patrol blood vessels as they respond to inflammation signals in tissues, such as exposure to viral products such as gp120, which can trigger chemokine production [72]. Upregulation of chemokines lead to movement of monocytes into various tissues and this process is thought to drive trafficking of monocytes through the blood–brain barrier. Studies also observed higher levels of HIV-1 DNA in activated inflammatory monocytes (CD14+/CD16+) than in non-activated monocytes [73]. Accumulating evidence shows that the route of CNS infection appears to involve circulating activated monocytes (CAM) [73]. Increase in peripheral activated monocyte subsets correlates with HIV disease progression and severity of neurocognitive impairment [62,74]. Research has demonstrated that individuals with HAD had higher levels of circulating activated CD14 monocytes and monocytes that co-express CD69, as compared to those without HAD. The finding supports the hypothesis that activated monocytes enter the brain and subsequently trigger neurotoxin production [75]. Individuals with HAD have a higher percentage of circulating activated monocytes/macrophages (M/MΦ), suggesting that HIV-1 DNA harboured in these reservoir cells is transported into the CNS by the same cells [73,76]. This evidence supports that accumulation of activated macrophages/microglia resulting from systemic immune activation trigger neurodegeneration in HAND [72,77]. It is clinically not feasible to sample brain tissue to determine macrophage/microglial viral DNA levels in clinical studies and routine diagnostics. Therefore, blood is the best option. Investigations using some animal models have shown that infected monocyte transmigration into the CNS appear to trigger gene expression of quiescent cells [61]. This finding raises the possibility that latent HIV harboured in peripheral monocytes may have clinical consequences upon BBB transmigration [60]. Viral DNA in blood monocytes in one study was significantly associated with eight of the nine cognitive domains that are frequently impaired in HAND [78,79]. Using magnetic resonance spectroscopy, it was demonstrated that neuronal injury and glial dysfunction was positively linked to peripheral CD14+ HIV DNA [80,81].

## 4. Quantitative PCR Assays to Detect Total HIV-1 DNA

There are a number of real-time PCR based assays to measure total HIV-1 DNA or cell associated DNA. These assays differ in extraction methods, PCR cycling conditions and amplification targets [82]. Some assays lack the sensitivity to quantify all the HIV-1 subtypes in the major (M) group, due to the high degree of genetic variability of the virus [83]. The genetic differences may result in DNA copy number variations due to probe and primer mismatches. The differences in PCR-based protocols for quantifying cell-associated HIV DNA has made it difficult to compare studies. Some of the confounding factors that result in non-specific variation in proviral load include input template quantity and quality, yields of the extraction process and enzymatic reactions [84]. Therefore, it is very important to prescribe the minimum requirements for a PCR-based assay to determine HIV proviral load. A requirement for quality PCR is that the extraction method produce high purity DNA that is free from haemoglobin, alcohol or high salt concentrations that may interfere with PCR amplification. Another critical requirement for determining HIV DNA proviral load in clinical samples is the assessment of the number of human cells per sample before DNA extraction [85]. The number of cells determined by flow cytometry is used to normalise HIV DNA in a sample [86]. Normalisers are used to ensure that target quantities from an equivalent amount of samples are comparable in absolute quantification, [73,87]. Data normalisation, which can be done by including an endogenous gene (reference gene) in the assay, is one of the essential key elements on the Minimum Information for Publication of Quantitative Real-Time PCR Experiments (MIQE) guidelines [88].

Normalisation is done to reduce non-biological variation as much as possible so that a true biological variation, explaining the phenomenon under investigation, is determined [84,89]. Housekeeping genes, such as Glyceraldehyde phosphate (GAPDH), β-globin, β-actin and Telomerase Reverse Transcriptase (TERT), determined by qPCR, were used for normalisation of HIV DNA to cell equivalents in some studies [28,31,73,90]. These PCR methods are more accurate than cell counts from cell sorting. The normaliser can also be used for quality control purposes since the numbers of cell inputted affect the sensitivity, as well indicating the presence of PCR inhibitors that might alter the sensitivity of the assay [91].

Malnati et al. in 2008, developed a universal HIV-1 group M-specific qPCR with a similar degree of accuracy and sensitivity across all viral subtypes. The designed primers and probe allow correct quantification of virtually all circulating HIV-1 strains. The universal assay amplifies part of the LTR-*gag* region that is highly conserved among all circulating group M HIV-1 subtypes [92]. The success of this group M-specific HIV-1 qPCR is based on the optimised design of primers and probes that ensure quantification of all the strains in the group. To obtain a standard for construction of a calibration curve, a fragment containing the conserved LTR-*gag* region was amplified from the T lymhoblastoid cell line using the optimised primers as described by Malnati. The fragment was then cloned into pCRII plasmid, from which serial dilutions were made to make standard curves. An additional qPCR was developed, in this assay, to measure the number of human cells initially present in a sample and to normalise the HIV-1 proviral DNA. This was done by measuring the copy number of a single copy human gene CCR5. The CCR5 assay permits the assessment of the sample quality as well as the number of cells. This protocol performs well if the input sample contains between 1000 and 150,000 cells per reaction. This robust method can quantify HIV-1 DNA from a crude lysate with the same degree of accuracy as with purified DNA. The advantage of a crude lysate is that it circumvents the nucleic acid extraction process, makes the assay take a shorter time and reduces the costs of the assay. The assay becomes suitable for large clinical trial cohorts.

Using cell lysate, Vandergeeten et al. [91] developed a nested real-time to measure total HIV DNA from diverse subtypes. The first round of PCR was pre-quantification duplex PCR with two sets of primers, one for the HIV-1 and the second set for the normaliser CD3 gene. The second round PCR was real qPCR performed separately for HIV quantification and for determination of cell input using the CD3 gene. The primers and probes for this assay, which target the conserved LTR-*gag* region, allows the precise quantification of total HIV DNA from subtypes, A, B, C, D, CRF01 A/E and CRF02_A/G, with the detection limit of three copies for all subtypes. The assay also showed that HIV DNA copies were detected with similar efficacy if the cell input ranges from 10,000 to 300,000. The assay clearly demonstrated the cell input sensitivity of HIV DNA quantification. 

Recently, a simple and reproducible qPCR assay for cell-associated HIV-1 DNA was developed targeting the conserved 3’ part of the *integrase* region of the pol gene with a sensitivity of three to five copies per million cells [82]. The 3’ end of the HIV-1 *pol* gene has been shown to be the most highly conserved region, meaning that primers and probes targeting this region should amplify efficiently the largest numbers of samples from HIV-1 infected people without mismatches. The sensitivity of this assay was determined by testing the limiting dilution of DNA standards in accordance with the Poisson distribution. The assay was able to detect three copies of HIV DNA standards in 80–100% of the reactions. Low detection limit (LOD) of three genome copies shows that the assay is very sensitive. Inhibition of HIV DNA PCR by excess nucleic acid was prevented by the dilution of the extract to ≤170 ng/μL. The nucleic acid extract was also diluted by 1:30 for CCR5 qPCR. The control of the nucleic acid input for both HIV DNA and the CCR5 normaliser is important to avoid underestimation of proviral load through inhibition. Many other PCR based assays are used in monitoring the HIV reservoir in clinical trials and HAND studies, but they differ in many ways including target region, normaliser, sample type, sample input quantity, standards, limit of detection and cycling conditions [26,31,82]. Table 1 shows selected methods used in qPCR for HIV DNA showing different parameters, including the ANRS commercial assay, which has been evaluated for inter-laboratory reproducibility.

## 5. Relevance of Total HIV DNA qPCR Assays to HAND

Many studies have evaluated and demonstrated the prognostic value of total HIV DNA measurement by qPCR as a marker of disease progression, independent of plasma viral load and CD4+ count [27,36,93,94]. High levels of HIV DNA were associated with rapid progression to AIDS and death in treatment naïve patients [41]. A suitable HIV DNA qPCR assay for HAND should target a clinically relevant marker and should be highly specific, sensitive, reproducible, cost-effective and easy to implement in clinical settings. Both replication-competent and defective forms of HIV DNA have been shown to be clinically relevant to neurocognitive impairment [44,60,61]. It is well understood that the different forms of HIV DNA co-exist in an infected cell, although their distribution varies depending on whether the cells are activated or latent [36,57,93,95]. Therefore, it is important to quantify all forms of HIV DNA, including the defective and unintegrated forms, and utilise them as surrogate biomarkers for HAND pathogenesis. It is also cost effective to have a qPCR that quantifies all the different forms of HIV DNA rather than separately quantifying integrated forms, 1-LTR or 2-LTR. Total HIV DNA qPCR assays can be applied to whole blood, PBMCs, monocytes or lymphocyte subsets and is easy to measure [31,34,42,96]. The assay quantifies all forms of HIV DNA that co-exist in infected cells that are involved in HIV pathogenesis [31,57]. Comparing measures such as 2LTR circles or integrated HIV DNA, total HIV DNA has the advantage of easy quantification, by standardised sensitive, real-time PCR methods [34]. It is suitable for analysing large samples with accuracy, requires a relatively small amount of blood and is not affected by freezing–thawing [96]. However, there is no commercial assay currently available for HIV DNA qPCR [41]. The above-described assays, including the ones that have been used on HAND studies, are a modification of commercial assays used in HIV RNA quantification [82,97]. Thus, an assessment of qPCR applied in HAND studies is required with regard to sensitivity and the sample types that were used in the assays.

## 6. HIV-1 DNA qPCR Used in HAND Studies

Application of qPCR and digital PCR to determine the association between HIV DNA levels in PBMC and its fractions, in the context of HAND, needs to consider both clinical and demographic variables of the study cohorts as well as the severity of HAND and performance characteristics of the assays. Using a very sensitive qPCR assay (LOD of 1–3 copies), it has been demonstrated that HIV DNA correlated with HAD in both HAART-naïve and HAART treated patients [73,80,81]. These studies also showed that activated monocytes HIV DNA levels in HAD patients were higher than in lymphocytes including the CD4+ cells. The sensitivity of the assay allows detection of low copies in suppressed patients and those who commence ART early in the course of HIV infection. Using a full range of neurocognitive disorders, the assay also showed that people with normal cognition had lower peripheral HIV DNA levels as compared to those with minor motor disorders and HAD [80]. Results of this study showed that the assay was sensitive enough to determine the association between circulating HIV DNA and different stages of HAND from normal cognition to the extreme forms of HAD. The HIV DNA determined by this assay, as a proposed biomarker, has been shown to be proportional to all three levels of neurocognitive function as well as different neuropsychological deficits, making it applicable for both screening and monitoring therapeutic interventions.

The HIV DNA assay targeting the *gag* region has shown that monocyte HIV DNA levels correlated with all three clinical categories of HAND (ANI, MND and HAD) in HAART treatment naïve patients [11]. The study further demonstrated association between monocyte HIV DNA and glial dysfunction, neuronal injury and CSF immune activation. The important aspect of this study demonstrates the link between both neurobehavioral and neuropathological aspects of HAND and circulating HIV DNA levels. However, there was no association between PBMC HIV DNA and HAND. Different HIV DNA PCR assays including droplet digital PCR used on HAND studies produced different results depending on clinical and demographic characteristics of study participants, as well as performance characteristics of the assays [42,51,80]. Table 2 shows selected PCR assays used in HAND studies with clinical data for study participants and performance characteristics of the assays.

## 7. Possible Use of Digital PCR to Detect HIV DNA in HAND

The amplification signal is logarithmic and the quantification is based on an external calibration or standard curve in the above described qPCR methods. A relatively newer technology called digital PCR (dPCR) offers simple, linear and digital quantification that is based on the number of positive and negative reactions using Poisson distribution [98]. Digital PCR uses limiting dilutions and sample partitioning into submicrolitre reactions. Each partition represents an isolated end point PCR [99]. The Poisson distribution of template molecule within these partitions is used to deduce the concentration of the target nucleic acid from frequency of negative to partitions. Digital PCR has been shown to be tolerant with sequence variations by higher robustness to primers and probe mismatches with target sequences, giving a superior advantage for HIV-1 DNA quantification and, as a result, dPCR has shown higher accuracy precision and reproducibility over qPCR.

Regarding detection and quantification of HIV proviral DNA in HAART suppressed patients, which are usually very low but relevant to HAND, dPCR can offer an alternative to qPCR based assays [51]. Most patients who are on long term ART, the proviral load ranges from 10 to 1000 copies of HIV DNA per million cells [25,99]. The higher sensitivity of dPCR compared to qPCR will be more relevant to the determination of low quantities of HIV DNA in monocytes in the periphery, which have been shown to be predictive of HAND and thought to be a major source of virus production in the brain [67]. Although dPCR has a great potential to be used as a valid alternative to qPCR, there is need to eliminate false positive droplets, which have been reported in negative template controls [100]. The other hurdle in the utility of dPCR for precise measurement of HIV DNA is the lack of a standardised automated method for determining threshold droplets. Threshold setting is very important to determine baseline fluorescence in order to distinguish between positive and negative droplet populations without bias [101]. There is also a need to work on the reduction of cost so that dPCR can be clinically accredited.

## 8. Conclusions

Classical virological and immunological parameters such as plasma viral load and CD4+ T-cell counts for HIV normally used to monitor the progression of disease cannot be used to measure and predict the onset of HAND. Most of the proposed markers of HAND are similar to the biomarkers of other neurocognitive disorders due to the similarities in the molecular mechanisms that are involved. The HIV DNA or cell-associated DNA (CAD) is detected in the peripheral blood of HAART suppressed patients and many studies have shown it to correlate with HAND. However, there is a need for an assay that is robust, sensitive and accurately quantifies HIV DNA. Total HIV DNA quantification by real-time PCR from unfractionated PBMCs is very simple, but HIV DNA from inflammatory CD16+ monocytes is linked to the pathogenesis of HAND and, therefore, more suitable as a marker. Regarding quantification of low level HIV DNA in HAART suppressed patients, dPCR offers a superior advantage, but issues to do with standardization of threshold and cost reduction need improvement before utilisation in diagnostic settings. Therefore, qPCR methods remain relevant now for clinical use since research has shown significant association between peripheral HIV DNA levels in cell compartments and severity of HAND. Additionally, evaluated commercial qPCR kits for HIV DNA load are now available. However, more data are required on T cell HIV DNA as an indicator of potential HAND-associated reservoirs in untreated patients.

## Figures and Tables

**Table 1:** Selected technologies used in quantitative HIV DNA real time PCR assays.

Assay	Normalisation	Unique Features and Advantages/Disadvantages	PCR Template Loading Quantity	LOD	Reference
Whole blood ANRS *LTR* real time PCR Method	HIV-DNA copies/µg converted to copies/million leucocytes	HIV DNA extracted from re-suspended whole blood cell pellets No separation of cells Its quicker and cost serving Normalisation using cell count/cytometry is not accurate	1 µg DNA, equivalent to 150,000 cells	1 Copy	[34]
LTR ANRS LTR real time PCR Method	Copies/million/PBMC	First assay for estimating HIV reservoir size to be evaluated for inter-laboratory reproducibility. The assay available as a commercial	1 µg DNA, equivalent to 150,000 cells	1 Copy	[34]
SYBR Green gag HIV DNA PCR	Copies/hundred thousand cells	Uses SYBR green fluorescence and amplifies a 142-*gag* fragment The assay is not sensitive especially in suppressed patients	300 ng, equivalent to 1,000,000 cells	50 Copies	[27]
Cross-Clade Ultrasensitive nested PCR	CD3 gene copy cell equivalents	Assay is performed on cell lysate, hence circumvents laborious nucleic acid extraction Allows quantification of HIV DNA A, B, C, D, CRF01_A/E, and CRF02_A/G HIV-1 subtypes, targeting highly conserved LTR-*gag* region. Cell input equivalence is accurately quantified by CD3 gene quantitative PCR. It has a disadvantage of 2 rounds of PCR	100,000 Cells	3 Copies	[91]
universal real-time PCR for group-M HIV-1 DNA	CCR5 gene copy cell equivalence	HIV-1 M-specific quantitative measures the HIV-1 Proviral DNA load in group M with a similar degree of sensitivity and accuracy across subtypes. Uses cell lysate as a template Predilution required if loading quantity is above 150,000 cells	1 µg per reaction, equivalent to 150,000 cells	1 Copy	[92]
Novel Assay for Total cell associated HIV-1 DNA	CCR5 gene copy cell equivalence	Sensitive Quantitative real time PCR for HIV-1 Cell associated DNA (CAD) targeting 3’ region of the *pol* gene. Assay involves enhanced nucleic extraction by ensuring adequate cell lysis through ultrasonic cell disruption	1.7 µg	3 Copies	[82]

**Table 2:** HIV DNA real time PCR assays applied in HAND studies.

Reference	Target	Sample	Clinical Data	Study Outcome	Advantage/Disadvantage
[73]	*gag*	PBMC	HAD patients on HAART 20–39 years and 50 years and above	Quantities of HIV DNA correlated with HAD HIV was higher in activated monocytes than CD14− in two patients	Very sensitive Quantitect Sybr Green PCR assay with detection limit of 1–3 copies and applicable suppressed patients and those with low cell PBMC or cell subset counts
[78]	*gag*	PBMC, CD14+, CD14−	15 HAART-naïve HAD patients and 15 Non demented patients Average age, 34.1	HIV DNA in PBMC was significantly higher in HAD non demented patients HIV DNA in five HAD patients was higher in CD14/CD16 than CD4	Very sensitive Quantitect Sybr Green PCR assay with detection limit of 1–3 copies and can be applied to suppressed patients and samples with low cell count
[31]	*pol*	PBMC	HIV Subtype B chronic patients on cART for six months CD4+ < 350/mL Median Age 56, 74	HIV DNA was associated with HAD and not Non demented forms of HAND	Very sensitive assay with one copy as LOD, hence suitable for supressed HAND patients PCR has a wide dynamic range from three copies to 300,000 copies High efficiencies for both HIV DNA and β-actin normaliser (93.7% and 86.6% respectively) -Assay restricted to detecting Subtype B
[42]	LTR-*gag*	CD14− and CD14+	ART naïve participants (median age, 32) with 17 impaired and 19 unimpaired Patients had higher HIV DNA in lymphocytes than monocytes	Strong association between HIV DNA levels in lymphocytes and HAND	The target region for amplification is highly conserved and the Universal PCR assay has been customised for subtype G and CRF02_AG. However, performance of the assays not detailed
[79]	gag	CD14+	12 HAART treated HAD and 15 non HAD Infected with HIV-1 CRF01_AE Median Age, 32	Baseline monocyte HIV DNA correlated with HIV DNA and 48 weeks after HAART. Monocyte HIV DNA level was below detection limit in all non-HAD patients after 48 weeks	Detection limit of the assay was 10 copies/10^6^ cells which is relatively high.
[11]	*gag*	CD14+PBMC	Treatment naïve with three clinical categories of HAND (ANI, MND, HAD) CD4+ <350/mL –CRF AE_01Mean Age, 35	No correlation between PBMC HIV DNA Before CD14+ enrichment, CD14+ was associated with HAND(ANI, MND, HAD) –CD14+ HIV DNA was associated with glial dysfunction, neuronal injury and CSF immune activation (neopterin)	Very sensitive assay with a limit of detection of one copy which is suitable for HAND suppressed patients
[51]	*pol*	PBMC	22–40 years and 50–71 years ART-supressed HIV subjects	Higher HIV DNA levels were associated with more severe neurocognitive impairment in older patients No association between HIV DNA and HAND in young adults.	Very sensitive droplet digital PCR with Limit of Detection (LOD) of one copy and more precise than qPCR due insensitivity to primer and probe mismatches absolute quantification, no external standards required
